# Comparative Proteome Analysis of Wheat Flag Leaves and Developing Grains Under Water Deficit

**DOI:** 10.3389/fpls.2018.00425

**Published:** 2018-04-10

**Authors:** Xiong Deng, Yue Liu, Xuexin Xu, Dongmiao Liu, Genrui Zhu, Xing Yan, Zhimin Wang, Yueming Yan

**Affiliations:** ^1^College of Life Sciences, Capital Normal University, Beijing, China; ^2^College of Agronomy and Biotechnology, China Agricultural University, Beijing, China; ^3^State Key Laboratory of Earth Surface Processes and Resource Ecology, College of Global Change and Earth System Science, Beijing Normal University, Beijing, China

**Keywords:** bread wheat, flag leaves, developing grains, 2D-DIGE, proteome, drought stress

## Abstract

In this study, we performed the first comparative proteomic analysis of wheat flag leaves and developing grains in response to drought stress. Drought stress caused a significant decrease in several important physiological and biochemical parameters and grain yield traits, particularly those related to photosynthesis and starch biosynthesis. In contrast, some key indicators related to drought stress were significantly increased, including malondialdehyde, soluble sugar, proline, glycine betaine, abscisic acid content, and peroxidase activity. Two-dimensional difference gel electrophoresis (2D-DIGE) identified 87 and 132 differentially accumulated protein (DAP) spots representing 66 and 105 unique proteins following exposure to drought stress in flag leaves and developing grains, respectively. The proteomes of the two organs varied markedly, and most DAPS were related to the oxidative stress response, photosynthesis and energy metabolism, and starch biosynthesis. In particular, DAPs in flag leaves mainly participated in photosynthesis while those in developing grains were primarily involved in carbon metabolism and the drought stress response. Western blotting and quantitative real-time polymerase chain reaction (qRT-PCR) further validated some key DAPs such as rubisco large subunit (RBSCL), ADP glucose pyrophosphorylase (AGPase), chaperonin 60 subunit alpha (CPN-60 alpha) and oxalate oxidase 2 (OxO 2). The potential functions of the identified DAPs revealed that a complex network synergistically regulates drought resistance during grain development. Our results from proteome perspective provide new insight into the molecular regulatory mechanisms used by different wheat organs to respond to drought stress.

## Introduction

Wheat (*Triticum aestivum* L.) is an extensively cultivated cereal crop base on its value as a staple food and protein source. Drought is one of the main abiotic stresses that limit yield in many crop species during grain filling. Global warming and climate change have exacerbated the effects of abiotic stresses on crop production; a temperature increase of 1°C can result in a decrease in yield of up to 10% (Lobell et al., 2011). Drought stress disrupts cellular homeostasis and gives rise to morphological, physiological, and molecular changes. In particular, drought stress disrupts photosynthesis and transfer of stored carbohydrates into grains during the crop flowering stage, which reduces grain number and weight (Richards et al., 2011). This reduction is exacerbated by stress at the early grain-filling stages (Stone and Nicholas, 1995). In addition, remobilization of stored carbon reserves in wheat is facilitated by water stress and water deficit during grain filling, which enhances plant senescence and accelerates grain filling (Yang et al., 2000, 2001). Therefore, it is important to explore the molecular mechanisms underlying the response of plants to drought stress to improve crop drought resistance and minimize yield loss.

The primary biological function of leaves is photosynthesis, which is the ultimate yield-limiting factor (Slafer et al., 1990). Wheat flag leaves have the highest photosynthetic efficiency of all leaves at later growth stages and serve as an important source of carbohydrate for grains, which contribute to wheat grain yield up to 41–43% (Araus and Tapia, 1987). Photosynthesis is particularly sensitive to water deficit. The foliar photosynthetic rate and relative water content (RWC) are decreased under drought stress (Lawlor and Cornic, 2002). Stomatal limitation is a major factor in the subdued photosynthesis seen under drought stress (Cornic, 2000). In addition, drought limits photosynthesis through metabolic impairment. The changes of cellular carbon metabolism are probably to take place early in the dehydration processes. Moreover, drought generally cuts down the carbon assimilation and utilization capacity of plants.

Wheat grain endosperm consists of about 70% starch and 14% proteins, which contribute to grain yield and quality (Johansson et al., 2001). These reserve substances are gradually accumulated during grain development and a lot of genes are involved in this progress (Yu et al., 2016). In higher plants, starch biosynthesis in the endosperm plants occurs within the amyloplast and involves at least four types of enzyme: AGPase, starch synthases (SS), branching enzymes, and debranching enzymes. Drought directly influences starch biosynthesis by reducing the activities of these related enzymes. In addition, photosynthesis provides the ingredient for starch biosynthesis; therefore, any disruption of photosynthesis impairs carbon metabolism and so reduces starch biosynthesis and grain yield.

Drought stress gives rise to a series of physiological and biochemical responses in plants; e.g., repression of cell growth and photosynthesis, stomatal closure, and activation of respiration. Plants also respond and acclimatize oneself to water deficit at the cellular and molecular levels; e.g., by accumulating reactive oxygen species (ROS) and proteins involved in drought tolerance. Under drought stress, plant root caps produce the hormone abscisic acid (ABA) to trigger a signaling cascade in guard cells that results in stomatal closure and decreases water loss (MacRobbie, 1998). This in turn suppresses cell growth, photosynthetic efficiency, and respiration (Shinozaki and Yamaguchi-Shinozaki, 2007; Budak et al., 2013). However, there is a lacking correlation between stomatal conductance and xylem ABA, but a superior correlation with leaf ABA (Henson et al., 1989; Ali et al., 2007). Thus, stomatal regulation in response to soil dryness is connected with ABA accumulation in leaf tissues, at least in wheat (Saradadevi et al., 2014).

Exposure of plants to adverse environmental conditions results in changes in detoxification pathways. Most of these changes can be regarded as the part of detoxification signaling. These include phospholipid hydrolysis, changes in the expression of late embryogenesis-abundant (*LEA*)/dehydrin-type genes, molecular chaperones, and proteinases, together with activation of enzymes involved in the generation and removal of ROS: singlet oxygen, superoxide radical (O_2_^-^), hydrogen peroxide (H_2_O_2_), and hydroxyl radical (OH) (Zhu, 2002; Drazkiewicz et al., 2007). Moreover, plants scavenge high levels of ROS by producing superoxide dismutase (SOD), catalase (CAT), and peroxidase (POD), enzymes involved in the ascorbate–glutathione (AsA–GSH) cycle, as well as other antioxidant compounds.

The molecular mechanism of drought responses and tolerance in plant species, including Arabidopsis (Reumann and Singhal, 2014), rice (Wan and Liu, 2008), soybean (Das et al., 2016), and napus (Koh et al., 2015), has been investigated using a proteomic approach. In wheat, only limited studies were reported on the proteome response to field drought stress during grain development (Mohsen et al., 2007, 2014; Gu et al., 2015). These studies have mainly concentrated on individual organs and so their results do not reflect any synergistic response mechanisms of different organs, particularly flag leaves and developing grains. In this study, we performed the first comparative proteomic analysis of wheat flag leaves and developing grains under field drought stress and analyzed their physiological and biochemical parameters, and yield traits. The results enhance our understanding of the regulatory networks of wheat flag leaves and developing grains in response to drought stress.

## Materials and Methods

### Wheat Materials, Field Drought Treatments, and Sampling

“Zhongmai 175” (*Triticum aestivum* L.), an elite Chinese winter wheat cultivar, was used in this study and planted at the experimental station of China Agricultural University (CAU), Wuqiao, Hebei Province (116°37′23″E and 37°16′02″N) during the 2014–2015 wheat growing season. The organic matter, total nitrogen, hydrolysable nitrogen, and available phosphorus and potassium levels in the topsoil (0–20 cm) of the experimental plots were 12.1 g kg^-1^, 1.0 g kg^-1^, 106.7 mg kg^-1^, 33.8 mg kg^-1^, and 183.4 mg kg^-1^, respectively. The level of precipitation in the wheat growing season is shown in Supplementary Figure S2A.

The field experiment involved two irrigation treatments: no irrigation after sowing (drought treatment group) and two irrigations after sowing (at jointing and anthesis, 75 mm of water each) as the control group. Each experimental plot was 8 m × 4 m with rows spaced at 0.16 m increments with three replications. One meter interval between plots was designed as an unirrigated zone to minimize the effects of adjacent plots. A flow meter was used to measure the amount of water applied. Soil samples were collected at 0.2 m increments to a depth of 2 m using a soil corer. Measurements were performed at the beginning of anthesis and at maturity. The soil water content was determined using the oven-drying method (Gardner, 1986). In addition, the determination of the soil relative water (SRWC) was based on Wang et al. (2014).

As a supplemental irrigation (Chu et al., 2016), before sowing the target relative soil water content of the 0–200 cm soil layer was 80% of the field capacity, and so the soil water content was irrigated to 80.5% of the field water capacity. Crop developmental stages were classified by the Zadoks scale (Zadoks et al., 1974). Plants were marked after flowering, and flag leaves as well as developing grains from five periods (10, 15, 20, 25, and 30 days post-anthesis, DPA) in three biological replicates were harvested. All collected samples were immediately transferred to liquid nitrogen for storage prior to analysis.

### Physiological and Biochemical Parameter Measurements

Plant, spikelet, and grain phenotypes in the control and drought treatment groups were assessed at the indicated developmental stages.

The LI-3100 area meter (Li-Cor, Inc., Lincoln, NE, United States) was used to measure the flag leaf area and length. The chlorophyll content and stomatal conductance of flag leaves were measured using a SPAD-502 Minolta chlorophyll meter (Spectrum Technologies, Plainfield, IL, United States). The above measurements were performed on 10 leaves per plot at 5-day intervals from 10 to 30 DPA.

Canopy temperature was measured multiple times during grain filling between 12:00 and 13:00 using a handheld thermometer (Reytek ST20XB; Reytek Corporation, Albuquerque, NM, United States). Canopy temperature depression (CTD) was computed as the difference between the air temperature at during measurement and canopy temperature, to account for fluctuations throughout the measurement period (Reynolds et al., 2001). The normalized difference vegetative index (NDVI) was determined using a portable spectroradiometer (GreenSeeker Handheld Crop Sensor; Trimble, Navigation Ltd., Sunnyvale, CA, United States). The sensor was held 60 cm above the canopy. NDVI was computed from measurements of light reflectance in the red and near-infrared (NIR) regions of the spectrum, as follows: (NIR – R)/(NIR + R), in which R is the reflectance in the red band and NIR is the reflectance in the NIR band (Reynolds et al., 2001).

The net photosynthesis rate (Pn) of flag leaves was measured at 5-day intervals from 10 to 30 DPA (from 9:00 AM to 11:00 AM) using an LI-6400 Portable Photosynthesis System (LI-COR Bioscience Inc., Lincoln, NE, United States) under artificial light (1,200 ± 50 μmol⋅m^-2^⋅s^-1^). The RWC, malondialdehyde (MDA) content, soluble sugar content, proline content, glycine betaine content, and POD activity in flag leaves were measured according to Lv et al. (2016). The sucrose synthase (SS) activity, AGPase activity, and total starch content of wheat grains were determined according to Zhen et al., 2017. ABA, indoleacetic acid (IAA), gibberellins (GA3), and zeatin riboside (ZR) levels in flag leaves were quantified by enzyme-linked immunosorbent assay (ELISA) according to Yang et al. (2004), with slight modifications. All measurements involved three biological replicates.

### Scanning Electron Microscopy

Grain ultrastructure was visualized by scanning electron microscopy (SEM) following our recent report (Chen et al., 2016).

### Protein Extraction, 2D-DIGE, and Image Analysis

The total albumin and globulin of flag leaves and developing grains were extracted according to Zhang et al. (2014) with slight modifications. Mixing pairs of Cy3- and Cy5-labeled protein samples with a Cy2-labeled internal standard were subjected to two-dimensional difference gel electrophoresis (2D-DIGE). The DIGE images were analyzed using DeCyder software (ver. 6.5; Amersham, Little Chalfont, United Kingdom). 2D-DIGE analysis was based on Rollins et al. (2013) and Cao et al. (2016). Details on the 2D-DIGE experiments for differentially accumulated protein (DAP) identification and expression analysis are listed in Supplementary Table S1. Protein labeling, 2D-DIGE, imaging, and image analysis were performed according to Bian et al. (2017) and Li et al. (2017), with minor modifications. Only those with significant and biological reproducible changes (abundance variation at least two-fold, Student’s *t*-test, *p* < 0.05) were considered to be DAP spots. Three biological replicates were used for all samples.

### Two-Dimensional Electrophoresis and Protein Identification by Tandem Mass Spectrometry

Two-dimensional electrophoresis (2-DE) was applied to separate DAP spots, and tandem mass spectrometry (MS/MS) analysis was used to identify DAP spots based on Cao et al. (2016). Proteins (600 μg) in 360 μL rehydration buffer (7 M urea, 2 M thiourea, 2% w/v 3-[(3-cholamidopropyl)dimethylammonio]-1-propanesulfonate (CHAPS), 0.2% bromophenol blue, 65 mM dithiothreitol (DTT) and 0.5% immobilized pH gradient (IPG) buffer) were loaded onto an 18 cm linear gradient IPG strip (GE Healthcare, Little Chalfont, United Kingdom) and separated by 2-DE. The ImageMaster 2D Platinum 7.0 (GE Healthcare, United States) was used to analyze the images and only those with significant and biological reproducible changes (abundance variation at least two-fold, Student’s *t*-test, *p* < 0.05) were considered to be DAP spots. We randomly collected the flag leaves and grains of 300 wheat plants from three experimental plots, respectively, mixed them, and randomly weighed three 1-g heavy leaves and grains for 2-DE and follow-up experiments.

After having excised the DAP spots from the 2-DE gels manually, transferred them to centrifuge tubes (2.0 mL) for digestion with trypsin as described by Lv et al. (2016). Spectra were obtained using an ABI 4800 Proteomics Analyzer matrix-assisted laser desorption/ionization time-of-flight/time-of-flight mass spectrometer (MALDI-TOF/TOF-MS) operating in result-dependent acquisition mode. The MS/MS spectra were searched against Viridiplantae (green plant) sequences in the non-redundant National Center for Biotechnology Information (NCBI) database and Triticum NCBI database using MASCOT software (ver. 2.1; Matrix Science, London, United Kingdom) with the following parameter settings: trypsin cleavage, one missed cleavage allowed, carbamidomethylation set as fixed modification, oxidation of methionines allowed as variable modification, peptide mass tolerance set to 100 ppm, and fragment tolerance set to ±0.3 Da. All searches were evaluated based on the significant scores obtained from MASCOT. The protein score CI% and total ion score CI% were both set to >95%, and a significance threshold of *p* < 0.05 was used.

### Bioinformatics Analysis

Venn diagram analysis of the identified DAP spots was performed using online software ‘Venny^1^.’ Protein function classification was based on the annotation from UniProt (Wang et al., 2015). The subcellular localization was predicted according to the integration of prediction results of the FUEL-mLoc Server^2^, WoLF PSORT^3^, CELLO version 2.5^4^, Plant-mPLoc^5^ and UniProtKB. Principal component analysis (PCA) was conducted in the R language and Environment for Statistical Computing (version 3.0.2, Auckland, New Zealand) (Valledor and Jorrín, 2011). Thirteen physiological and biochemical parameter of flag leaves at different developmental stages were homogenized by (X-mean value)/(standard deviation) and then carried out PCA analysis (SPSS v. 19, SPSS Inc., Chicago, IL, United States). whole data sets and DAP spot data sets in flag leaves and developing grains of wheat, at five developmental stages in the control and drought treatment groups, were analyzed by PCA. A cluster analysis of differentially abundant proteins was performed using Cluster software version 3.0. Euclidean distances and Ward’s criteria were used in the analysis. Cluster results were visualized using Java TreeView software^6^.

### Western Blotting

Sodium dodecyl sulfate-polyacrylamide gel electrophoresis (SDS-PAGE) was performed according to Yan et al. (2003). Proteins (30 μg) in buffer solution were loaded onto a 12% gel and resolved at 15 mA for 2.5 h. The gels were subjected to Western blotting according to our previous report (Chen et al., 2016). The anti-Rubisco large subunit (AS03 037) and anti-AGPase (AS11 1739) antibodies were from Agrisera (Stockholm, Sweden).

### Total mRNA Extraction and qRT-PCR

Quantitative real-time-polymerase chain reaction (qRT-PCR) was performed to determine the dynamic transcript levels of key DAPs. Flag leaf and grain samples from eight developmental periods (8, 10, 13, 15, 17, 20, 25, and 30 DPA) were ground into fine powder in liquid nitrogen. Then, total RNA was isolated from each sample using TRIzol reagent (Invitrogen, Carlsbad, CA, United States), and reverse transcription reactions were performed using a PrimeScript^®^ RT Reagent Kit with gDNA Eraser (TaKaRa, Shiga, Japan) according to the manufacturer’s instructions. Gene-specific primers were designed using Primer3Plus^7^ (Untergasser et al., 2007) and their specificities were checked by melting curve analysis of RT-PCR products and the corresponding bands in agarose gels. The primer sequences for the qRT-PCR assays are listed in Supplementary Table S4. Ubiquitin was used as the reference gene. Transcript levels were quantified using a CFX96 Real-Time PCR Detection System (Bio-Rad, Hercules, CA, United States) with the intercalating dye SYBR-green following the 2(-Delta Delta C(T)) method (Livak and Schmittgen, 2001). qRT-PCR was performed as described previously (Bian et al., 2017). The optimal parameters yielded a correlation coefficient (*R*^2^) of 0.994–0.999 and PCR amplification efficiency (E) of 90–110% (Supplementary Figures S7A,B). Three biological replicates were performed for each sample.

## Results

### Physiological and Biochemical Parameters and Agronomic Traits

During the 2014–2015 winter wheat growing season in Wuqiao, total precipitation was 128 mm (Supplementary Figure S2A), which is lower than the annual mean (130–180 mm). The changes in relative soil water content at a 2 m depth in the control and drought treatment groups are shown in Supplementary Figures S2B,C. According to the grade of agricultural drought (GB/T 32136-2015), severe drought occurred in the 0–60 cm soil layer, and mild drought in the 60–120 cm soil layer at anthesis in the drought treatment group. At maturity, severe drought occurred throughout the 0–100 cm soil layer. Plant growth period was advanced, leaves turned yellow, wheat ears were smaller and the plants were shorter under drought stress (Supplementary Figures S1A–C). The CTD at the middle-late grain filling stage was increased and the NDVI at the whole grain filling stage was decreased under drought stress (Supplementary Figure S2E). The drought treatment group also exhibited significant changes in physiological and biochemical characteristics, main agronomic traits and yield performance.

The physiological and biochemical parameters of flag leaves (Supplementary Figures S3A–M) and developing grains (Supplementary Figures S3N,O) differed significantly between the control and drought treatment groups. In leaves, the total chlorophyll content, RWC, Pn, stomatal conductance, and GA_3_ level decreased gradually from 10 to 30 DPA in both groups, but were significantly reduced by drought stress at different developmental stages. The MDA, soluble sugar, proline and glycine betaine contents increased significantly as leaf development progressed in the drought treatment group. The ABA, IAA, and ZR contents and POD activity in flag leaves exhibited an increase-decrease expression tendency during grain development in the control group, but displayed various expression patterns in the drought treatment group. The ABA content was increased at 10 and 15 DPA, but decreased significantly at 30 DPA (Supplementary Figure S3G). The IAA content in the drought treatment group was significantly higher at 15 and 20 DPA and significantly lower at 25 and 30 DPA, compared to that in the control group. The ZR content decreased throughout grain development, and POD activity was significantly increased at 10, 20, and 30 DPA. SS and AGPase activities were decreased significantly in the drought treatment group (Supplementary Figures S3N,O).

**FIGURE 1:**
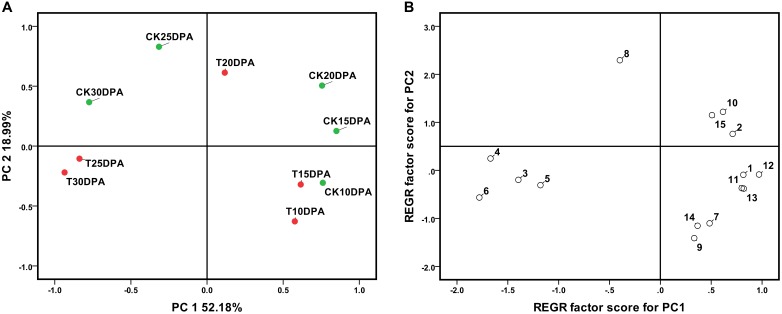
The principal component analysis **(A)** and the principal component regression **(B)** of 13 leaf parameter at different developmental stages of Zhongmai 175. 1: Chlorophyll; 2: RWC; 3: MDA content; 4: Soluble sugar content; 5: Proline content; 6: Glycine betaine content; 7: ABA content; 8: IAA content; 9: GA3 content; 10: ZR content; 11: POD activity; 12: Pn; 13: Stomatal conductance. CK and T indicate the control group (irrigation at jointing and anthesis stages) and drought treatment group (no-irrigation after sowing), respectively.

Further PCA showed that PC1 and PC2 could correctly separate the samples. Spots loadings analysis indicated that spots which showed a higher correlation with PC1 were parameters related to developmental stages, PC1 was named as development stage. Similarly, the spots with a higher correlation with PC2 were parameters related to treatment, PC2 was named as treatment. Both drought treatment and development stages had significant effects on leaf physiological and biochemical parameters as revealed by their distinct grouping in the PCA plot (**Figure 1A**). Principal component regression analysis illustrated that spot 7 (ABA contents), spot 8 (IAA contents), spot 9 (GA_3_ contents), and spot 10 (ZR contents) show a higher correlation with REGR factor score for PC2 (**Figure 1B**), suggesting that these parameters are more sensitive to water deficit and could be considered as major indicators of the response to the drought treatment.

Analyses of major agronomic and yield traits showed that drought treatment significantly decreased flag leaf width and area, plant height, spike number (10,000/ha), grain number per spike, and grain starch content; and increased the number of infertile spikelets, ultimately resulting in a 19.23% decrease in grain yield (Supplementary Figures S2F–H). Drought treatment increased starch biosynthesis at early grain developmental stages, but significantly decreased starch content from 65.37% (control group) to 60.68% (drought treatment group) at grain maturation. Starch content increased by 13.53% from 30 to 45 DPA after drought stress, but increased by 18.98% over the same period in the control group (Supplementary Figure S2D).

### Ultrastructure of Developing Grains Under Drought Stress

**FIGURE 2:**
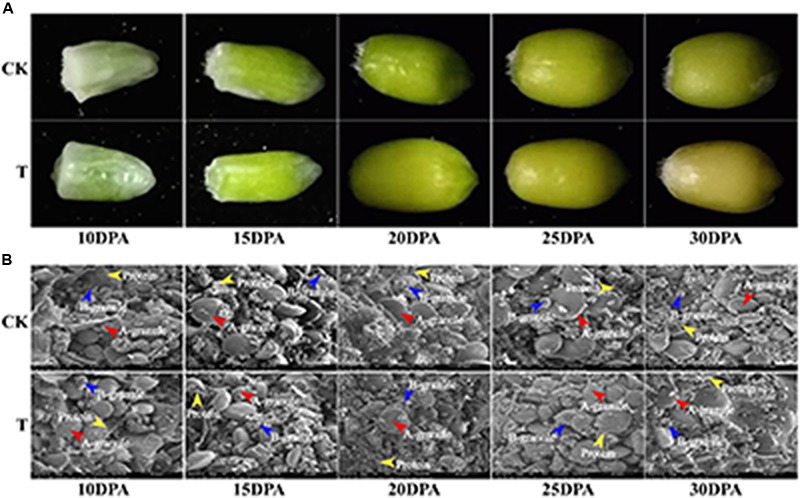
Grain phenotype and ultrastructure changes under drought stress. **(A)** Grain phenotype changes at different developmental stages in both groups. **(B)** SEM images of developing grains from five periods in CK and drought treatment group. The scale bar is 20 μm. **(A,B)** Starch granules are marked with red and blue arrows, respectively, and the protein bodies are marked with yellow arrows.

Grain sizes in both groups gradually increased from flowering to maturity, but the grain size and rate of development differed significantly. The control group generally had a larger grain size, earlier grain filling and longer grain-filling period than the drought treatment group (**Figure 2A**). The dynamic ultrastructural changes of developing endosperm observed by SEM showed that A and B granules in both groups were initiated at 10 DPA, and gradually increased in size as grain development progressed. However, the drought treatment group generally had fewer and smaller A and B granules, but more protein bodies, compared to the control group at all developmental stages (**Figure 2B**). Therefore, starch granule formation was significantly inhibited during middle and late grain developmental stages, which is consistent with the changes in starch content and SS activity.

### DAPs in Flag Leaves and Developing Grains Under Drought Stress

Differentially accumulated proteins in flag leaves and grains at five developmental stages were identified by 2D-DIGE. In total, 95 and 141 DAP spots were identified in flag leaves and grains, respectively (Supplementary Figure S4). Subsequently, 2-DE was used to separate proteins. All of the DAP spots identified by 2D-DIGE could be reproducibly detected and well matched at different developmental stages by 2-DE in both flag leaves and grains (Supplementary Figures S5A,B). Next, the DAPs were manually excised from gels, digested by trypsin, and subjected to MALDI-TOF/TOF-MS analysis. Finally, 87 (91.58%) DAP spots representing 66 unique DAPs in flag leaves, and 132 (93.62%) representing 105 unique DAPs in developing grains, were successfully identified. Their detailed information and peptide sequences are listed in Supplementary Tables S2A,B, S3A,B. The number of proteins gradually decreased in flag leaves and increased in developing grains as grain development progressed (Supplementary Figure S5).

**FIGURE 3:**
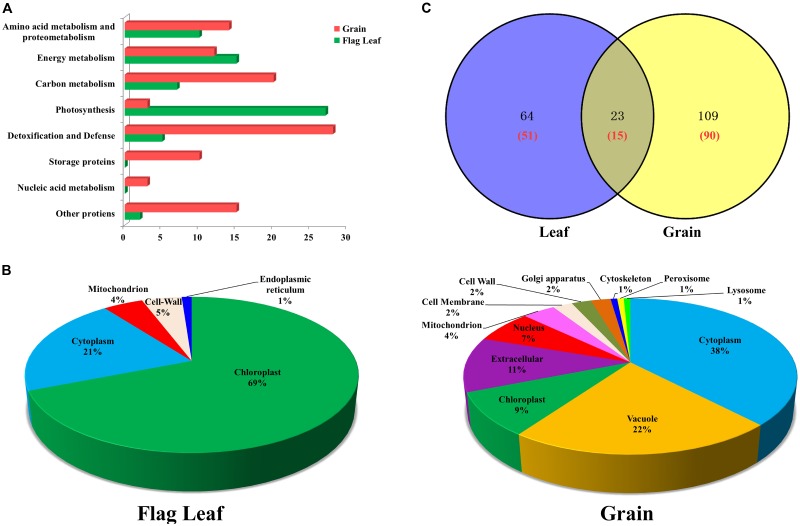
Functional classification, subcellular localization and Venn diagram analysis of DAPs from flag leaves and developing grains of Zhongmai 175. **(A)** Functional classification of DAPs from flag leaves and grains. **(B)** Subcellular localization of DAPs from flag leaves and developing grains. **(C)** Venn diagram analysis of DAP spots in flag leaves and developing grains under drought stress. The red number represents the number of unique protein species identified.

The 66 unique DAPs in flag leaves were classified into the following six functional categories (**Figure 3A**): photosynthesis, energy metabolism, amino acid metabolism and proteometabolism, carbon metabolism, detoxification, and defense and other proteins. The DAPs in grains were classified into eight functional categories (**Figure 3A**), principally detoxification and defense, carbon metabolism, energy metabolism, amino acid metabolism, and storage proteins. The largest two functional categories in flag leaves and developing grains were photosynthesis (40.91%)/energy metabolism (22.73%), and detoxification/defense (26.67%) and carbon metabolism (19.05%), respectively. Therefore, drought stress affected the levels of mainly photosynthesis and energy metabolism-related proteins in leaves and carbon metabolism and stress-related proteins in grains.

Subcellular localization prediction showed that 69% of the DAPs in leaves were localized in the chloroplast, followed by cytoplasm (21%), cell wall (5%), mitochondria (4%), and endoplasmic reticulum (1%) (**Figure 3B**). Similarly, DAPs in developing grains were distributed among 12 subcellular structures, principally in the cytoplasm (38%), vacuole (22%), and extracellular space (11%) (**Figure 3B**). The majority of enzymes participating in photosynthesis were located in chloroplast, and those participating in carbohydrate metabolism and detoxification, and defense, were located in the cytoplasm. Stress-related proteins were located mainly in peroxisomes, and most storage proteins were present in vacuoles and the extracellular space (Supplementary Tables S2A, S3A).

### Differential Proteome Analysis of Flag Leaves and Developing Grains Under Drought Stress

The number of DAP spots and their relationships are shown as Venn diagrams in **Figure 3C**. Among them, 23 DAP spots (11.73%) corresponding to 15 unique proteins were present in both organs, while 64 DAP spots (32.66%) corresponding to 51 unique proteins and 109 DAP spots (55.61%) corresponding to 90 unique proteins were specifically expressed in flag leaves and developing grains, respectively (**Figure 3A**). Therefore, developing grains harbored a greater number of DAPs than flag leaves.

**FIGURE 4:**
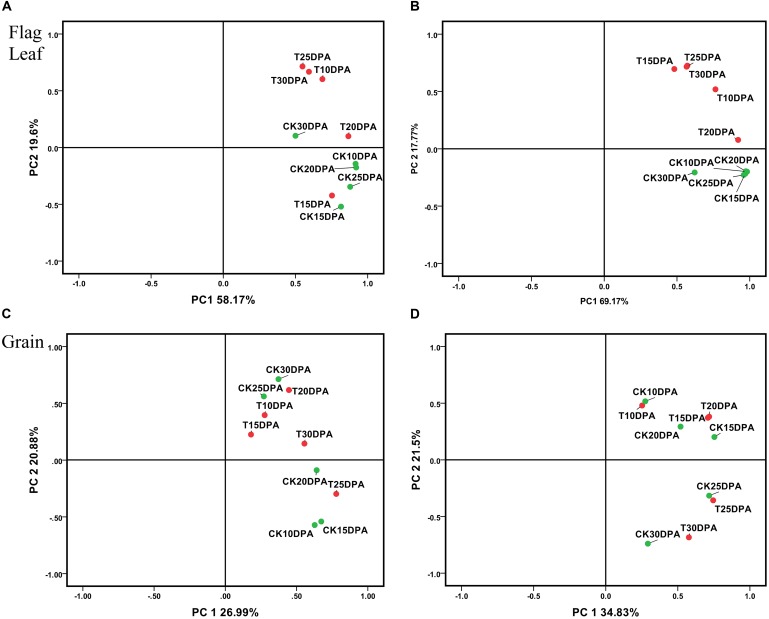
Principal component analysis (PCA) of all spots and DAP spot data sets from flag leaves and developing grains of Zhongmai 175. **(A)** PCA of all spots from flag leaves; **(B)** PCA of DAP spots from flag leaves; **(C)** PCA of all spots in developing grains; **(D)** PCA of DAP spots in developing grains.

All spots (537 in flag leaf and 650 in grain) and DAP spot (87 in flag leaf and 132 in grain) data sets were subjected to PCA to identify affected protein species, outliers, and clusters (Kristiansen et al., 2010; Luis et al., 2010; Valledor and Jorrín, 2011). The employment of these components, plotting PC1 and PC2, allowed the effective separation of samples into their original groups (**Figure 4**), and the plot structure was not greatly different between whole and DAP spots data sets. But the sum of the plotting PC1 and PC2 value from DAP spot data sets was greater than whole data sets both in flag leaf and grain (**Figures 4A–D**), which reflects the strong selection force that was applied to the original data set. As shown in **Figure 4B**, the spots in flag leaves which show a higher loading with PC2 were proteins related to treatment, PC2 was named as treatment. However, in **Figure 4D**, the spots in developing grains showing a higher loading with PC2 were proteins related to developmental stages, PC2 was named as developmental stages. Drought treatment and development stages, respectively, had significant effects on flag leaf and grain as revealed by their distinct grouping in the PCA plot, indicating that the proteome of flag leaves is more sensitive to drought stress than that of developing grains.

**FIGURE 5:**
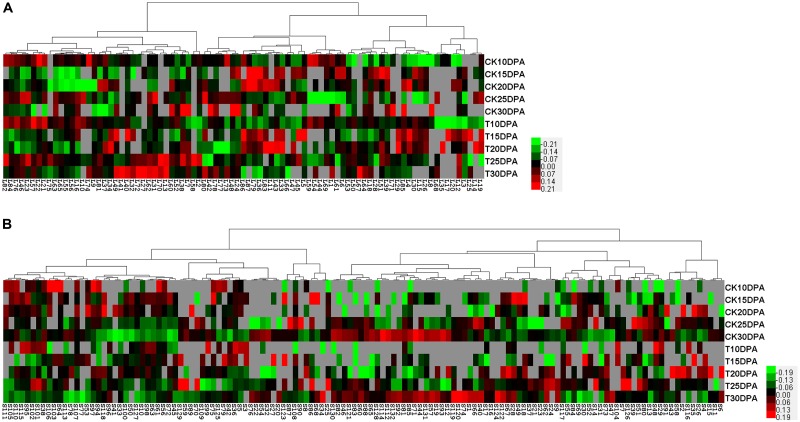
Protein expression clustering analysis of DAP spots from 2-DE maps of flag leaves and developing grains. **(A)** Hierarchical clustering of DAP spots from flag leaves; **(B)** Hierarchical clustering of DAP spots from developing grains. Each column represents samples from control and drought treatment groups. Each row displays the change of a DAP spot using color-coding based on the relative ratio.

To visualize coordinately regulated DAP spots, we performed a hierarchical cluster analysis to evaluate the changes in protein levels due to drought treatment. Two hierarchical clusters corresponding to flag leaves (**Figure 5A**) and developing grains (**Figure 5B**) were constructed. The DAP spots from flag leaves and developing grains were classified into four and five expression types, respectively. In flag leaves, pattern I proteins tended to be down-regulated. These proteins are mainly involved in photosynthesis. Pattern II proteins were mainly related to energy metabolism with an up- and down-regulation. In contrast, pattern III proteins, which were mainly involved in carbohydrate metabolism, were down- and then up-regulated. Pattern IV proteins were mainly related to the stress response and were up-regulated. In developing grains, pattern I proteins were mainly related to carbohydrate metabolism. Pattern II proteins mainly related to protein metabolism. Pattern III proteins were involved mainly in protein and nucleic acid metabolism, while pattern IV proteins were primarily involved in the stress response and energy metabolism. Pattern V proteins were up-, then down-, and then up-regulated, and most were globulins.

### Transcription Expression Profiles of Important DAP Genes

We selected 11 and 13 key DAPs in flag leaves and developing grains, respectively, and evaluated their dynamic expression changes in transcriptional level by qRT-PCR. The levels of all of the selected proteins differed significantly under drought stress, and were closely related to detoxification and defense (L41, L56, G2, G9, G13 G21, G84, and G94), photosynthesis (L2, L34, L36, and G37), energy metabolism (L5, L27, L42, L62, G78, and G101), carbohydrate metabolism (L21, G51, G64, and G123) and amino acid metabolism and proteometabolism (L11 and S119) (Supplementary Figure S6 and **Table 1**). These DAP genes displayed five primary expression patterns: up, down, up–down, down–up–down, and up–down–up–down. The transcript and protein levels of seven DAPs (L34, L36, L41, G51, G101, G119, and G123) showed high consistency, and those of six DAPs (L56, L62, G2, G13, G21, and G84) showed a similar trend. The transcript and protein levels of the remaining 11 DAPs (L2, L5, L11, L21, L27, L42, G9, G37, G64, G78, and G94) showed poor consistency, possibly due to post-translational modifications (Guo et al., 2012a). These results are generally consistent with previous reports (Ge et al., 2012; Jiang et al., 2012; Bian et al., 2017).

**Table 1:** Representative differentially accumulated proteins (DAPs) identified by MALDI-TOF/TOF-MS in flag leaves and developing grains of Zhongmai 175 under drought stress.

Spot no.	Protein name	Accession no.	Protein PI/MW	Protein score	Peptide count	Average %vol. ratio 10:15:20:25:30 (DPA)^∗^	*p*-Value	Subcellular localization
**Fifteen DAPs identified both in flag leaf and grain**								
L2/G37	Ribulose-1,5-bisphosphate carboxylase/oxygenase small subunit	gi|11990897	8.80/19.45	325	19	1:0.7:1.5:1:0.9	0.023	Chloroplast
L6/G45	5-Methyltetrahydropteroyltriglutamate-homocysteine methyltransferase	gi|473993302	5.74/88.5	1040	35	1:0.7:0.4:0.9:0.9	0.021	Cytoplasm
L7/G42	Putative aconitate hydratase	gi|473765331	5.66/93.86	614	31	1:0.3:0.6:2.2:1.1	0.022	Cytoplasm
L9/G78	Fructose-bisphosphate aldolase, cytoplasmic isozyme 1	gi|473936969	8.55/69.36	606	24	1:0.7:1.2:0.2:0.3	0.019	Cytoplasm
L14/G3	Ribulose-1,5-bisphosphate carboxylase/oxygenase large subunit	gi|667754420	6.04/52.7	415	20	1:0.4:0.7:0.7:0.7	0.026	Chloroplast
L27/G41	Enolase	gi|461744058	5.49/48.1	630	33	1:1.1:1.1:1.3:0.4	0.018	Cytoplasm
L41/G84	Dehydroascorbate reductase	gi|28192421	5.88/23.5	620	15	1:1.8:1.5:1.7:1.4	0.041	Cytoplasm
L52/G119	Methionine synthase 1 enzyme	gi|68655495	5.74/84.9	391	27	1:0.6:2.9:1.9:2.6	0.032	Cytoplasm
L22/G73	ATP synthase subunit	gi|285014508	8.18/39.7	255	20	1:1.7:2.4:1.9:1.3	0.043	Chloroplast
L86/G111	Elongation factor 2	gi|473786548	5.85/93.72	608	43	1:0.4:0.7:0.7:0.7	0.029	Cytoplasm
L62/G103	Isocitrate dehydrogenase (NADP)	gi|326494166	5.99/46.2	552	22	1:0.7:1.7:1.5:1.7	0.022	Chloroplast
L55/G87	Triosephosphate isomerase	gi|11124572	5.38/27.0	458	11	1:2.1:2.7:2.1:1.3	0.022	Cytoplasm
L17/G72	Fructose-1,6-biphosphate aldolase	gi|820943672	5.94/42	490	17	1:1.4:4.5:5.3:4.3	0.035	Cytoplasm
L32/G51	adenosine diphosphate glucose pyrophosphatase	gi|21322655	5.68/21.8	180	3	1:0.7:1.4:0.9:0.8	0.039	Cell-wall
L21/G79	Glyceraldehyde-3-phosphate dehydrogenase B	gi|473912215	6.03/46.9	442	14	1:1.5:10.4:6.2:14.1	0.029	Chloroplast
**Carbon metabolism**								
L24	Isopentenyl-diphosphate delta-isomerase II	gi|473943783	5.4/22.1	359	11	1:1.2:1.6:0.7:0.6	0.031	Chloroplast
G51	ADP-glucose pyrophosophorylase preprotein	gi|21680	8.7/33.06	124	2	1:0.31:0.15:0.11:0.17	0.026	Chloroplast
G62	Sucrose synthase type 2	gi|3393044	6.17/93.06	809	31	1:1.94:0.67:0.54:1.17	0.002	Cytoplasm
G99	Beta-amylase	gi|32400764	8.6/31.1	434	13	1:0.5:0.6:0.5:3.34	0.021	Mitochondrion
G98	Phosphoglucomutase	gi|18076790	5.66/62.98	476	18	1:1.16:1.06:0.2:0.71	0.019	Cytoplasm
G120	Alpha-glucan phosphorylase, H isozyme, expressed	gi|300681424	7.60/93.8	601	36	1:0.27:0.47:0.34:0.21	0.048	Cytoplasm
G130	Beta-D-glucan exohydrolase	gi|20259685	6.86/67.71	168	15	1:0.27:0.17:0.31:0.41	0.025	Lysosome
G131	Beta-glucanase	gi|600857	8.71/35.3	150	4	1:0.39:0.44:0.24:2.76	0.033	Cell-wall
G4	UDP-glycosyltransferase 73C5	gi|473759878	5.03/36.2	47	9	1:1.55:2.76:3.07:3.56	0.038	Cell-Membrane
G66	Aldose reductase	gi|475492917	6.51/35.63	1030	24	1:0.33:0.06:0.04:0.09	0.037	Cytoplasm
G97	Pyrophosphate—fructose 6-phosphate	gi|475604217	5.97/60.69	229	22	1:0.44:0.71:0.32:2.46	0.011	Cell-Wall
**Photosynthesis (the main function of leaves)**								
L10	Oxygen-evolving enhancer protein 1	gi|474352688	5.75/34.4	326	18	1:0.7:1.2:0.4:0.4	0.028	Chloroplast
L16	Phosphoribulokinase	gi|21839	5.84/45	380	21	1:1.2:3.6:3.1:0.7	0.035	Chloroplast
L20	Pyruvate, phosphate dikinase 1	gi|305691147	5.71/10.38	491	39	1:3:2.5:15.8:7.6	0.018	Chloroplast
L23	Chlorophyll a-b binding protein 8	gi|474121685	8.69/29.3	111	5	1:0.8:1.1:1.8:1.5	0.041	Chloroplast
L31	ATP synthase subunit beta, chloroplastic	gi|474022890	5.21/36.1	370	22	1:1.7:5.7:3:4.5	0.037	Chloroplast
L34	RuBisCO large subunit-binding protein subunit alpha	gi|474113969	5.17/65.3	869	34	1:2:1.9:1.9:2.3	0.035	Chloroplast
L44	33 kDa oxygen evolving protein of photosystem II	gi|21844	8.73/34.9	476	8	1:5.5:1.6:2.2:2.5	0.034	Chloroplast
L66	psbP domain-containing protein 6, chloroplastic	gi|326509981	7.71/29.4	279	7	1:1.4:4.2:1.9:2.3	0.037	Chloroplast
L71	Cytochrome b6-f complex iron-sulfur subunit, chloroplastic petC	gi|32394644	8.47/23.71	444	16	1:0.7:0.5:9.7:1.8	0.027	Chloroplast
L79	ATP-dependent Clp protease ATP-binding subunit clpA-like protein	gi|474241774	5.16/103.2	383	38	1:0.4:0.3:0.3:0.4	0.02	Chloroplast
G1	Pyruvate, phosphate dikinase 1	gi|474023061	5.66/122	491	39	1:3:2.5:15.8:7.6	0.018	Chloroplast
**Detoxification and defense**						
G11	Group 3 late embryogenesis abundant protein, partial	gi|170692	5.01/33.3	173	13	1:0.75:0.51:0.26:0.58	0.041	Cytoplasm
G13	Peroxidase	gi|290350668	8.14/38.8	95	5	1:2.68:1.88:0.6:8.32	0.037	Vacuole
G21	Oxalate oxidase 2	gi|474156730	4.98/30.9	97	5	1:1.37:1.02:0.41:0.61	0.021	Cell-wall
G25	Peroxidase 1	gi|300087071	8.14/38.8	340	13	1:0.39:0.44:2.17:0.47	0.032	Vacuole
G27	Heat shock protein 101	gi|4558484	5.95/101.1	741	39	1:0.98:0.68:0.15:0.42	0.023	Nucleus
G39	L-Ascorbate peroxidase 1	gi|474311703	5.85/27.4	238	12	1:2.54:1.13:1.34:1.85	0.046	Cytoplasm
G59	Superoxide dismutase	gi|226897529	5.71/15.3	124	6	1:0.34:0.14:0.46:1.98	0.009	Cytoplasm
G69	Peroxiredoxin-2C	gi|474145957	5.15/17.37	118	6	1:1.55:1:0.64:0.78	0.022	Cytoplasm
G85	Glutathione S-transferase	gi|5923877	5.79/23.61	200	4	1:0.6:2.08:0.53:1.04	0.015	Cytoplasm
G94	Catalase isozyme 1	gi|474292610	6.83/71.16	426	17	1:0.83:0.7:0.18:1.74	0.029	Peroxisome
G9	Serpin 1	gi|224589266	5.44/43.1	490	18	1:2.2:1.7:0.68:2.05	0.011	Extracellular
G15	Serpin-N3.2	gi|379060943	5.18/43	278	13	1:0.51:1.74:0.45:0.74	0.021	Extracellular
G22	Serpin-Z2B	gi|473793747	6.03/45.1	385	18	1:2.21:3.02:1.97:2.8	0.028	Chloroplast
G26	Serpin	gi|871551	5.6/43.1	72	6	1:0.71:0.85:0.31:0.58	0.047	Extracellular
G14	WCI proteinase inhibitor, partial	gi|20798981	7.42/12.9	128	3	1:0.61:1.38:2.91:4.04	0.026	Extracellular
G2	Alpha amylase inhibitor protein	gi|38098487	7.44/18.2	119	7	1:0.87:0.53:0.21:0.71	0.031	Extracellular
L80	Polyphenol oxidase	gi|296034254	5.88/63.68	257	14	1:2.1:4.3:2.7:2.9	0.044	Chloroplast
L26	9-*cis*-epoxycarotenoid dioxygenase	gi|765529848	6.11/67.5	376	21	1:1.7:1.5:2:1.3	0.021	Chloroplast
L56	Ferredoxin-NADP(H) oxidoreductase	gi|20302471	8.29/39.2	97	14	1:0.9:2.3:1.7:0.8	0.016	Chloroplast


*^∗^Abundance changes of DAP spots under water-deficit corresponding to the control.*

### Verification of Two Key DAPs by Western Blotting

**FIGURE 6:**
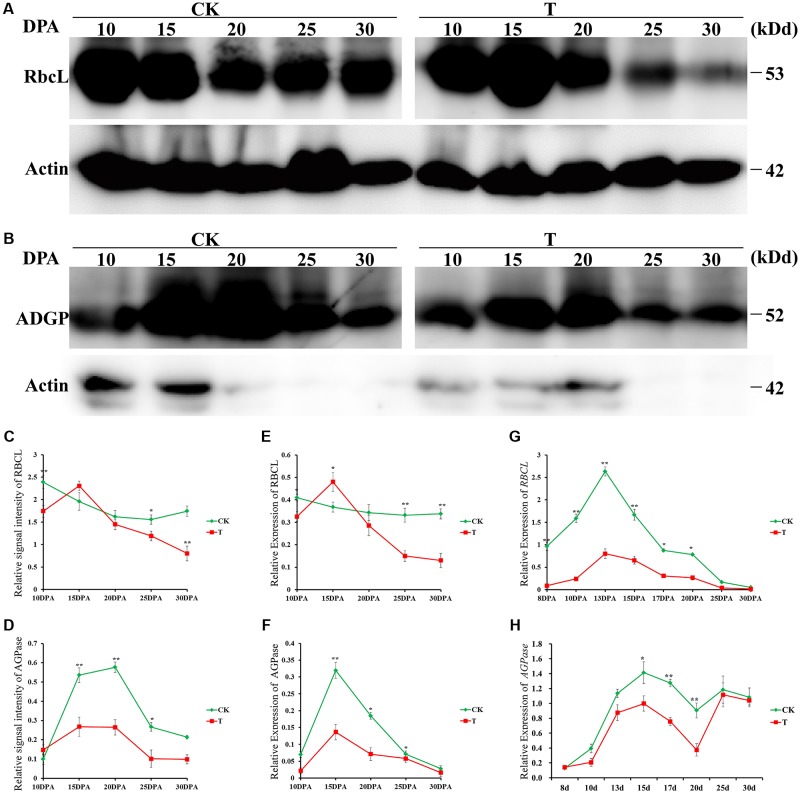
Western blotting verification of two key DAPs in response to drought stress. Immunoblot analysis of flag leaf RBSCL protein **(A)** and developing grain AGPase protein **(B)** at different developmental stages in the control and drought treatment groups by using anti-RBSCL and anti-AGPase antibody, respectively. Equal protein loading was confirmed by immunoblotting with an antibody against rice actin. The line chart represents quantification of the RBSCL bands **(C)** and AGPase band **(D)** by ImageJ. RBSCL and AGPase protein levels are expressed as a ratio of RBSCL to actin. The dynamic accumulation profiles of flag leaf RBSCL protein **(E)** and developing grain AGPase protein **(F)** were detected by 2-DE. The transcription level changes of flag leaf *RBSCL* **(G)** and developing grain *AGPase* **(H)** were detected by qRT-PCR. Error bars indicate standard errors of three biological replicates. Asterisks indicate ^∗^*p* < 0.05 and ^∗∗^*p* < 0.01 in Student’s *t*-test analysis.

To further verify the reliability of our proteomic dataset, Western blotting was performed to verify the proteome results of two key DAPs: ribulose-1,5-bisphosphate carboxylase/oxygenase large subunit (RBSCL, L14) in flag leaf and AGPase (G51) in grain; the levels of both were significantly different between the drought treatment and control groups (**Figures 6A,B**). Quantitative evaluation results of the RBSCL and AGPase bands done using ImageJ software (NIH, Bethesda, MD, United States) (**Figures 6C,D**) showed a significant decrease, consistent with the proteomics (**Figures 6E,F**), and transcript level (**Figures 6G,H**) data.

## Discussion

### Oxidative Stress Response

As a major abiotic stress, drought limits seriously wheat growth and yield. During their evolution, plants acquired mechanisms to respond to drought stress, an important example of which is the oxidative stress response. Plant endogenous hormones are closely related to plant growth and development, and play important roles in oxidative stress. In this study, the ABA, IAA, GA_3_, and ZR contents in leaves changed significantly in response to drought stress. In particular, the ABA content increased at early developmental stages (10 and 15 DPA) in flag leaves, which likely enhanced their drought resistance. ABA prevents the loss of water in plants by inducing production of H_2_O_2_, which activates Ca^2+^ channels and stomatal closure (Pei et al., 2000). In addition, IAA content increased significantly at 15 and 20 DPA, probably because that drought stress accelerates plant life and shortens growth period, but it decreased observably with the increase of soil drought degree and the prolongation of drought stress, consistent with the previous report (Liu et al., 2005). Leaf ZR content decreased significantly in drought group at all developmental stages. Similar results were also reported in spruce roots: ZR content decreased significantly in response to drought stress and ZR in the leaves mainly come from the roots (Ao and Wang, 2011).

Reactive oxygen species accumulate in plants subjected to drought stress. These ROSs function as important regulators of many biological processes, including stress responses, hormone signaling, cell growth, and development (Pei et al., 2000; Mittler et al., 2004; Bailey-Serres and Mittler, 2006; Fujita et al., 2006; Guo et al., 2012b). H_2_O_2_ activates phospholipid signaling (Kovtun et al., 2000; Grant et al., 2000; Desikan et al., 2001), which regulates stress tolerance in part by modulating the expression of stress-responsive genes, such as *LEA* (Zhu, 2002). In this study, group 3 LEAs were up-regulated in the drought treatment group at late developmental stages (25 and 30 DPA). LEA proteins are important in plants, as they are participated in abiotic stress tolerance, specifically dehydration and cold stresses (Wang et al., 2012). Group 3 LEAs of grains are reportedly intrinsically disordered and exist as random coils in solution at normal temperatures and water potentials, whereas potentially possess the propensity to assume helical conformations and act as molecular shields. This may increase its mechanical strength, in a manner similar to intermediate filaments, under drought stress (Wise and Tunnacliffe, 2004; Wang et al., 2012).

Under drought stress, plants experience oxidative stress due to an imbalance in the generation and removal of ROS, but are equipped with an antioxidant system to mitigate this (Zhang and Kirkham, 1994; Drazkiewicz et al., 2007). In this study, we identified nine enzymes associated with antioxidant stress in flag leaves and developing grains (Supplementary Tables S2A, S3A). SOD was up-regulated at 20, 25, and 30 DPA in grains under drought stress. SOD catalyzes the dismutation of superoxide anion radical (O_2_^-^) to H_2_O_2_ and O_2_ (Smirnoff, 1993). H_2_O_2_ is required for the ABA pathway, modulates the expression of stress-responsive genes, and is removed through the AsA–GSH cycle. AsA and GSH are not consumed during the AsA–GSH cycle, but they participate in cyclic transfer of reducing equivalents, which involves four enzymes and consumes H_2_O_2_ to generate H_2_O using electrons derived from NAD(P)H (Noctor and Foyer, 1998). In this study, we identified two of these enzymes: L-ascorbate peroxidase 1 (an APX) and DHAR. APX uses two molecules of AsA to reduce H_2_O_2_ to water, with concomitant generation of two molecules of monodehydroascorbate (MDHA), which is converted to AsA and dehydroascorbate (DHA) (Noctor and Foyer, 1998) during the response to drought stress. DHA is reduced to AsA by DHAR, using GSH as the reducing substrate (Foyer and Halliwell, 1976). This reaction generates glutathione disulfide (GSSG), which is in turn re-reduced to GSH by NADPH in a reaction catalyzed by GR. In this study, L-ascorbate peroxidase 1 was significantly increased to three-fold at 15 DPA in the drought treatment group, which could significantly improve the removal efficiency of H_2_O_2_ and maintain strongly the AsA–GSH dynamic balance. Glutathione transferases (GSTs) are involved in many biotic and abiotic interactions of plants with their environment. Drought-associated oxidative stress up-regulates the expression of *GST8* to counteract the effect of higher ROS production in stressed plants (Bianchi et al., 2002). Here, glutathione S-transferase was significantly up-regulated at 10 and 15 DPA under drought stress, likely to counteract the effect of higher ROS production under drought stress. PODs and CATs catalyze the conversion of H_2_O_2_ to H_2_O and molecular oxygen. Expression of the genes encoding these enzymes was increased or unchanged in the early phase of drought, and then a decrease with further increase in magnitude of water stress (Zhang and Kirkham, 1994). Similarly, the POD and CAT levels increased dramatically in grains during the early phase of drought treatment (10 and 15 DPA).

### Effect of Drought on Photosynthesis and Energy Metabolism Regulation

Photosynthesis is one of the key metabolic processes affected by drought stress. The foliar photosynthetic rate and leaf water potential are decreased under drought stress (Lawlor and Cornic, 2002). Under drought conditions, photosynthesis is reduced due to stomatal limitation and metabolic impairment, the former of which is the major determinant of reduced photosynthesis under drought stress (Cornic, 2000). Our data indicated that five parameters associated with stomatal limitation (leaf chlorophyll content, leaf RWC, net Pn, stomatal conductance, and leaf area) were significantly affected by drought stress (**Figure 1**). This likely decreased the internal CO_2_ concentration and inhibited photosynthesis.

We identified several DAPs associated with metabolic impairment, including a series of ribulose-1,5-bisphosphate carboxylase/oxygenase (Rubisco) proteins. The rate of photosynthesis in higher plants is dependent on the activity of Rubisco (Chaitanya et al., 2002; Parry et al., 2002). The Rubisco large and small subunits were down-regulated in flag leaves, but up-regulated in developing grains, under drought stress. In addition, pyruvate phosphate dikinase 1 (PPDK1) plays an important role in concentrating CO_2_ around Rubisco in the C4 pathway (Brown et al., 2005), and was up-regulated at 15 DPA in grains. Leaves are the major photosynthetic organs of wheat, but the presence of chloroplasts in the early grains indicates active photosynthesis. Indeed, developing wheat grains have a specific C4 photosynthesis (Rangan et al., 2016; Bachir et al., 2017; Henry et al., 2017). The C4 photosynthesis pathway has higher photosynthetic efficiency than the C3 pathway. Thus, the drought-mediated increase in photosynthesis in developing wheat grains may promote drought resistance.

Chlorophyll-binding proteins (CBPs) have diverse functions in light-harvesting and photoprotection (Bian et al., 2017). The LI818 family of CBPs plays a role in the stress response (45). In this study, chlorophyll a-b binding protein 8 was up-regulated under drought stress. This is in agreement with a previous report (Bian et al., 2017), and suggests that the photosynthesis light reaction was active under drought stress.

Plants require large numbers of proteins involved in carbohydrate metabolism and energy metabolism to maintain normal growth and development under stress conditions (Hossain and Komatsu, 2012). In this study, numerous proteins associated with energy metabolism were identified in leaves and developing grains, but with different expression patterns. This suggests that energy metabolism is regulated differently in leaves and grains under drought conditions. Under drought stress, four DAPs involved in glycolysis (fructose-bisphosphate aldolase, enolase, triosephosphate isomerase, and glyceraldehyde-3-phosphate dehydrogenase) were up-regulated at 10 DPA and then down-regulated rapidly in flag leaves. However, these proteins were up-regulated in grains at all developmental stages, with the exception of 10 DPA. Leaves are more sensitive to drought stress, likely due to suppression of glycolysis by stomatal closure. In addition, aconitate hydratase and isocitrate dehydrogenase (NADP), which are required for the tricarboxylic acid (TCA) cycle, were up-regulated at all developmental stages in flag leaf, but down-regulated in developing grains, under drought stress. We speculate that when subjected to drought, plants must increase TCA cycle activity in leaves and developing grains to provide sufficient ATP for physiological activities. Moreover, the ATP content and ATP/ADP ratio were markedly increased in spring wheat plants under drought conditions, indicating that up-regulation of the energy supply is important for drought stress response (Chen et al., 2004). Furthermore, starch synthesis was significantly increased at the late stages of grain development, likely related to enhanced TCA cycle and ATP synthase activity to increase ATP production.

### Regulation of Starch Biosynthesis Under Drought Stress

Photosynthesis provides triosephosphate for starch biosynthesis during early grain developmental stages (Tschiersch et al., 2010). Intermediates of the pentose phosphate pathway in the form of triose phosphates are released from chloroplasts for sucrose biosynthesis. Sucrose could be transported to the endosperm to participate in starch biosynthesis. We identified several key enzymes related to starch biosynthesis, including AGPase, SS 2 and phosphoglucomutase (PGM), in this study. AGPase catalyzes the first committed step of the starch biosynthetic pathway and converts glucose 1-phosphate and ATP to ADPG and pyrophosphate. AGPase was down-regulated at both the protein and transcript levels under drought stress (**Figure 6**), in agreement with a previous report (Jiang et al., 2012). On arrival in the cytosol of endosperm cells, sucrose is metabolized by sucrose synthase (Tomlinson and Denyer, 2003), which catalyzes starch synthesis by transferring the glucosyl moiety of ADP glucose to the non-reducing end of an existing α-1,4-glucan chain. We found that SS 2 activity was decreased markedly by drought stress. PGM catalyzes the interconversion of glucose-1-phosphate (G1P) and glucose-6-phosphate (G6P), with glucose 1,6-bisphosphate (G16BP) as a cofactor (Ray et al., 1983). In plant tissues, PGM is present in the cytosol and the plastid (Mühlbach and Schnarrenberger, 1978; Popova et al., 1998), and the cytosolic PGM reaction is important in the partitioning of carbon among starch synthesis pathways. According to our results, cytosolic PGM was up-regulated at 10 DPA and down-regulated at other time points under drought stress conditions. The plant growth period was advanced by drought, which led to up-regulation of PGM at the early stages and accelerated starch biosynthesis. At the later stages of grain development, drought stress resulted in downregulation of PGM, and consequently reduced starch biosynthesis and grain yield.

### A Putative Metabolic Pathway of Wheat Flag Leaves and Developing Grains in Response to Drought Stress

**FIGURE 7:**
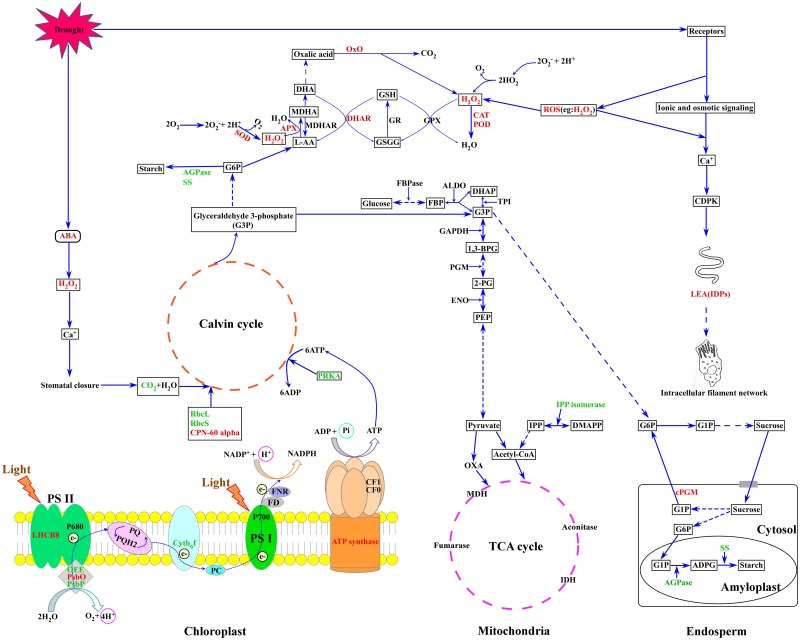
A putative metabolic pathway of drought stress responses in flag leaves and developing grains of Zhongmai 175. RbcL, ribulose-1,5-bisphosphate carboxylase/oxygenase large subunit; RbcS, ribulose-1,5-bisphosphate carboxylase/oxygenase small subunit; CPN-60 alpha, chaperonin 60 subunit alpha; PsbO, 33 kDa oxygen evolving protein of photosystem II; OEE, oxygen-evolving enhancer protein; LHCB 8, chlorophyll a-b binding protein 8; Cytb6-f, cytochrome b6-f complex iron-sulfur subunit; PRKA, phosphoribulokinase; SS, sucrose synthase; AGPase, ADP glucose pyrophosphorylase; SOD, superoxide dismutase; CAT, catalase; POD, peroxidase; APX, ascorbate peroxidase; DHAR, dehydroascorbate reductase; LEA, late embryogenesis abundant; PGM, phosphoglycerate mutase; PPO, polyphenol oxidase; OxO, oxalate oxidase. The red font represents up-regulated expression, and the green font represents down-regulated expression.

Based on our results and previous reports, a putative metabolic pathway that regulates drought resistance in wheat flag leaves and developing grains is proposed (**Figure 7**). In plants subjected to drought stress, ROS accumulation leads to an elevation of intracellular Ca^2+^ concentration, CDPK activation and triggering of signaling cascades that regulate the expression of stress-responsive genes. ROS inflicts oxidative stress, leading to activation of antioxidant systems. The increase in ABA content caused by drought stress induces the production of H_2_O_2_, which activates Ca^2+^ channels, resulting in stomatal closure. Subsequently, the internal CO_2_ concentration decreases and total photosynthetic metabolism is inhibited. In addition, drought stress reduced the expression and activities of enzymes involved in the photosynthetic carbon reduction cycle. Drought inhibited starch granule formation and starch biosynthesis by suppressing photosynthesis and starch biosynthesis-related enzymes, ultimately resulting in decreased grain weight and yield.

## Conclusion

Drought resulted in significant decreases in physiological and biochemical parameters related to photosynthesis and starch biosynthesis, as well as grain weight and yield. Comparative proteome analysis identified 87 DAPs in flag leaves and 132 DAPs in developing grains under drought stress conditions. DAPs from flag leaves were mainly involved in photosynthesis while those in developing grains mainly participated in carbon metabolism and drought stress response. DAPs associated with the oxidative stress response, mainly present in the developing grains were generally significantly up-regulated, while most of the DAPs related to photosynthesis in flag leaves and starch biosynthesis in developing grains were significantly down-regulated. Most of the DAPs associated with energy metabolism were down-regulated in flag leaves but up-regulated in developing grains. When subjected to drought, the response of flag leaves was more sensitive and rapid than that of grains. Drought significantly inhibited photosynthesis in leaves and carbon metabolism in grains, which could be responsible for the significant decrease in starch biosynthesis and grain yield. Plants respond to drought-induced oxidative stress by up-regulating production of antioxidant enzymes and those involved in the AsA–GSH cycle. Therefore, wheat flag leaves and developing grains respond to drought stress by modulating the expression of large numbers of genes whose products have diverse functions.

## Author Contributions

XD, YL, and XX performed most of the experiments, data analysis, and wrote the paper. DL performed part of the experiments and data collection. GZ performed Western blotting. XY, ZW, and YY designed and supervised the experiments.

## Conflict of Interest Statement

The authors declare that the research was conducted in the absence of any commercial or financial relationships that could be construed as a potential conflict of interest.

## Footnotes

1 http://bioinfogp.cnb.csic.es/tools/venny/

2 http://bioinfo.eie.polyu.edu.hk/FUEL-mLoc/

3 http://www.genscript.com/wolf-psort.html

4 http://cello.life.nctu.edu.tw/

5 http://www.csbio.sjtu.edu.cn/bioinf/plant-multi/

6 http://sourceforge.net/projects/jtreeview/

7 http://www.bioinformatics.nl/cgi-bin/primer3plus/primer3plus.cgi
