# Supplementary material for: Comparative Proteome Analysis of Wheat Flag Leaves and Developing Grains Under Water Deficit
Source: Front Plant Sci. 2018 Apr 10;9:425. doi: 10.3389/fpls.2018.00425 (PMC5902686; doi:10.3389/fpls.2018.00425)
Supplement: Supplementary file 1 [file Image_1.pdf]

Comparative Proteome Analysis of Wheat Flag Leaves and Developing Grains under Water Deficit

Xiong Deng[1,4], Yue Liu[1,4], Xuexin Xu[3,4], Dongmiao Liu[1], Genrui Zhu[1], Xing Yan[2*], Zhimin Wang[3*] and Yueming Yan[1*]

Figure S1. Morphological changes of bread wheat cultivar Zhongmai 175 under drought stress. **(A)** Wheat field performance at 25 and 30 DPA. **(B)** Plant height. **(C)** Spikes.

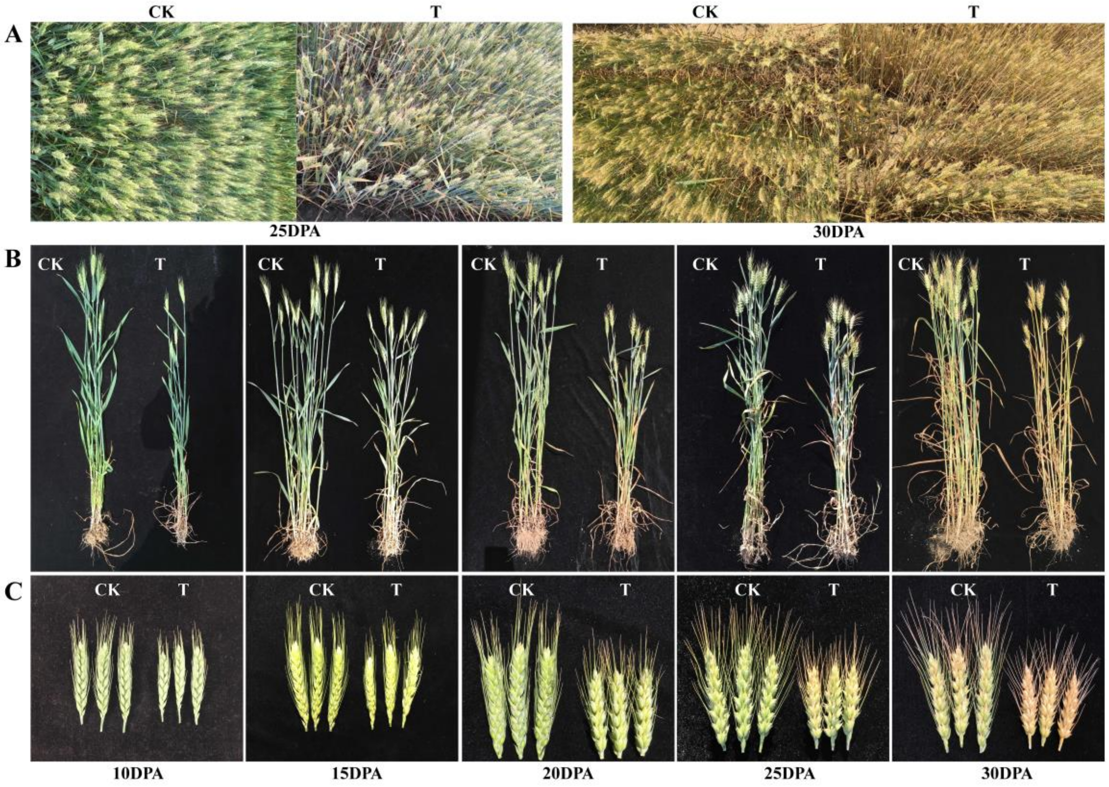

Figure S2. Rainfall, soil relative water content and agronomic trait changes under drought stress. **(A)** Rainfall in Wuqiao at 2014-2015 winter wheat growing season. **(B)** Soil relative water content (SRWC) at anthesis. **(C)** SRWC at maturity stage. **(D)** Starch content changes at different developmental stages under drought stress. **(E)** CTD and NDVI changes at the early filling and middle-late stages under drought stress. **(F)** Average area of 30 pieces flag leaves. **(G)** Average length of 30 pieces flag leaves. **(H)** Average width of 30 pieces flag leaves. CK and T indicate the control group (irrigation at jointing and anthesis stages) and drought treatment group (no-irrigation after sowing), respectively. Error bars indicate standard errors of three

biological replicates. Statistically significant differences compared to the control were calculated based on an independent Student's t-tests: *$p < 0.05$; **$p < 0.01$.

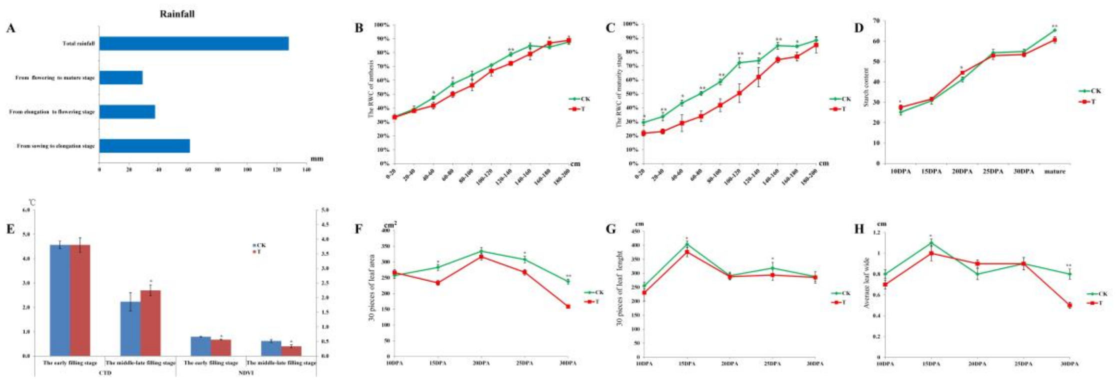

Figure S3. Physiological and biochemical parameter changes of flag leaves **(A-M)** and grains **(N-O)** at different developmental stages of Zhongmai 175 under drought stress. **(A)** Chlorophyll content; **(B)** Relative water content (RWC); **(C)** Malondialdehyde (MDA) content; **(D)** Soluble sugar content; **(E)** Proline content; **(F)** Glycine betaine content; **(G)** Abscisic acid (ABA) content; **(H)** Indoleacetic acid (IAA) content; **(I)** Gibberellin acid 3 (GA3) content; **(J)** Zeatin riboside (ZR) content; **(K)** POD activity; **(L)** Net photosynthesis rate. **(M)** Stomatal conductance. **(N)** Sucrose synthase (SS) activity; **(O)** ADP glucose pyrophosphorylase (AGPase) activity.

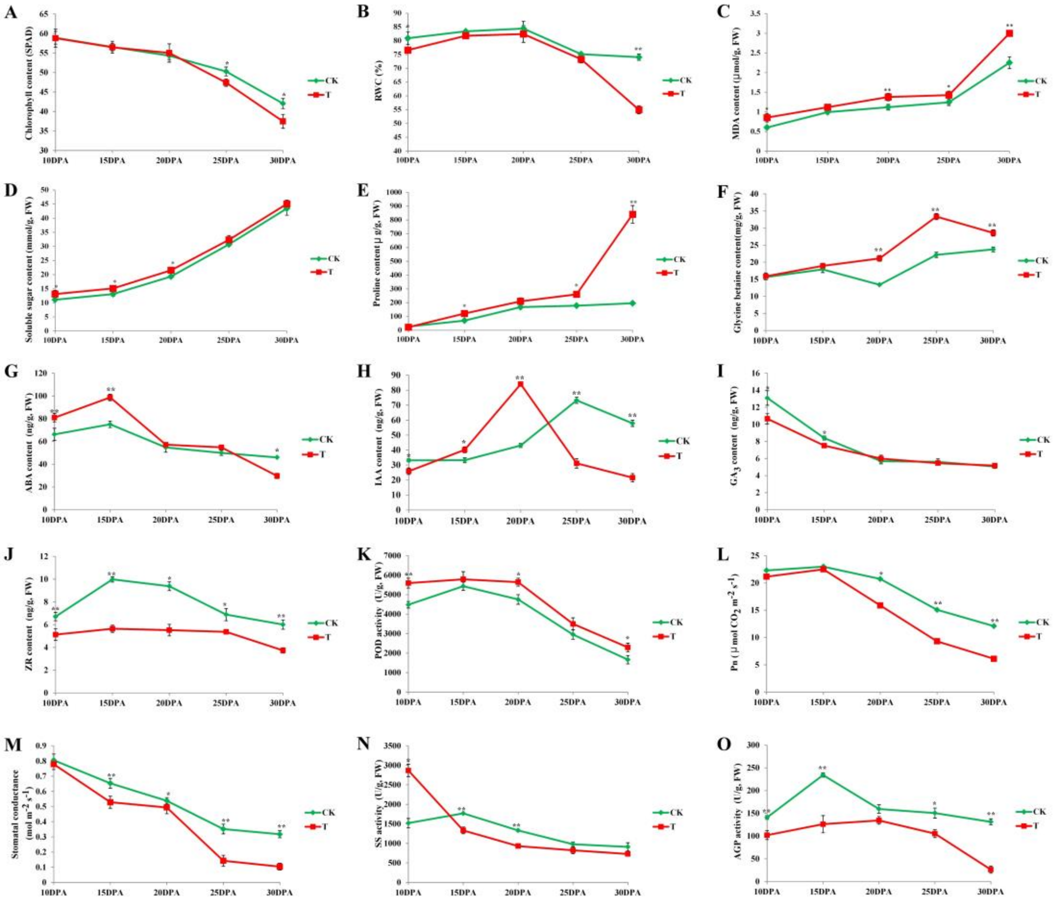

Figure S4. 2D-DIGE images of flag leaves and developing grains in Zhongmai 175 under drought stress. **(A)** Flag leaf gel 1; **(B)** Flag leaf gel 8; **(C)** Grain gel 16; **(D)** Grain gel 24. Numbered lines indicate differentially accumulated protein (DAP) spots identified by MALDI-TOF/TOF-MS with significant accumulation changes under drought stress.

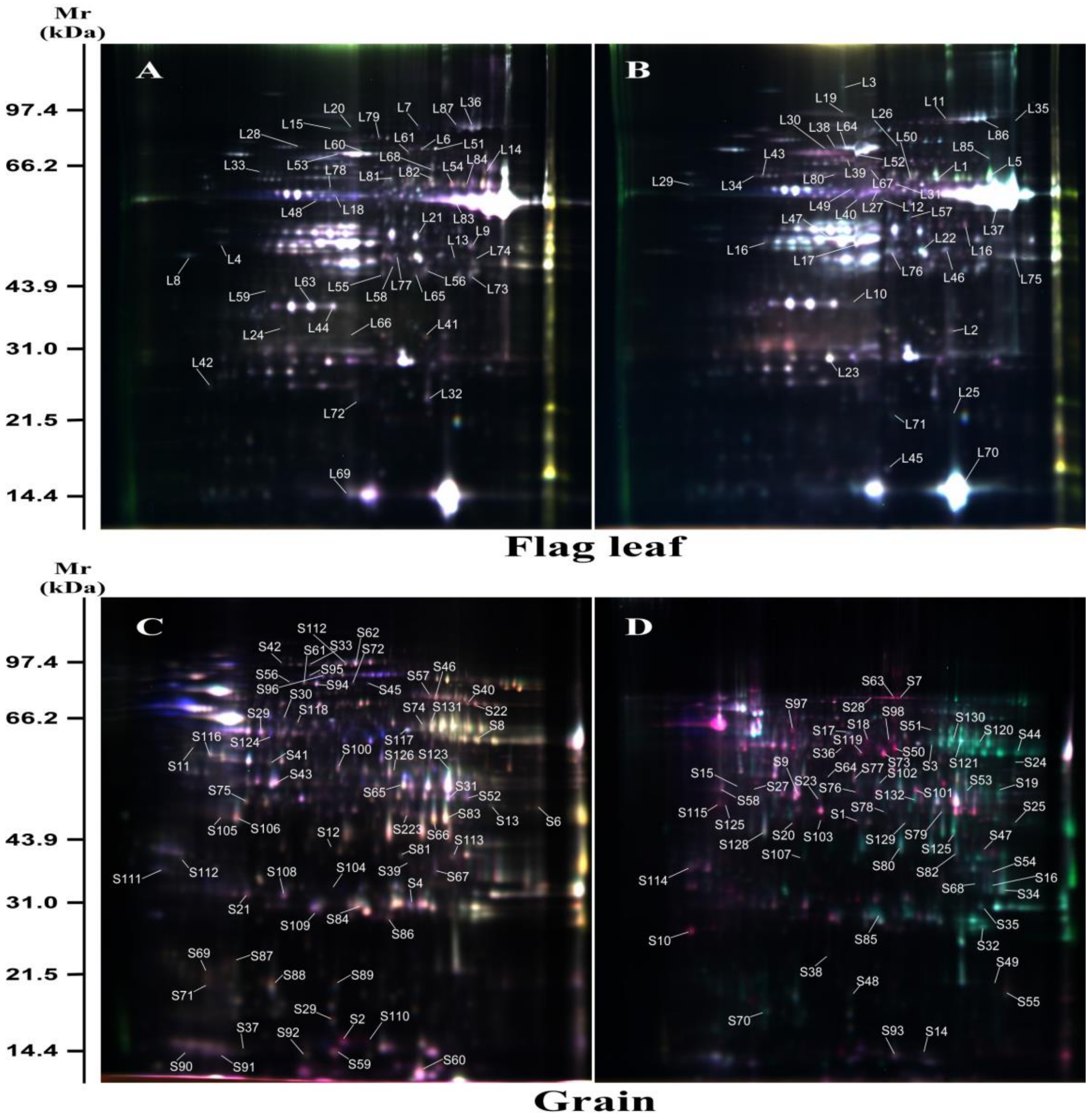

Figure S5 (A) Two-dimensional electrophoresis (2-DE) maps of flag leaf proteome in Zhongmai 175 under drought stress. A total of 87 DAP spots screened out from 2-DE maps of flag leaves are highlighted. **(B)** Two-dimensional electrophoresis (2-DE) maps of developing grain proteome in Zhongmai 175 under drought stress. A total of 132 DAP spots screened out from 2-DE maps of grains are highlighted.

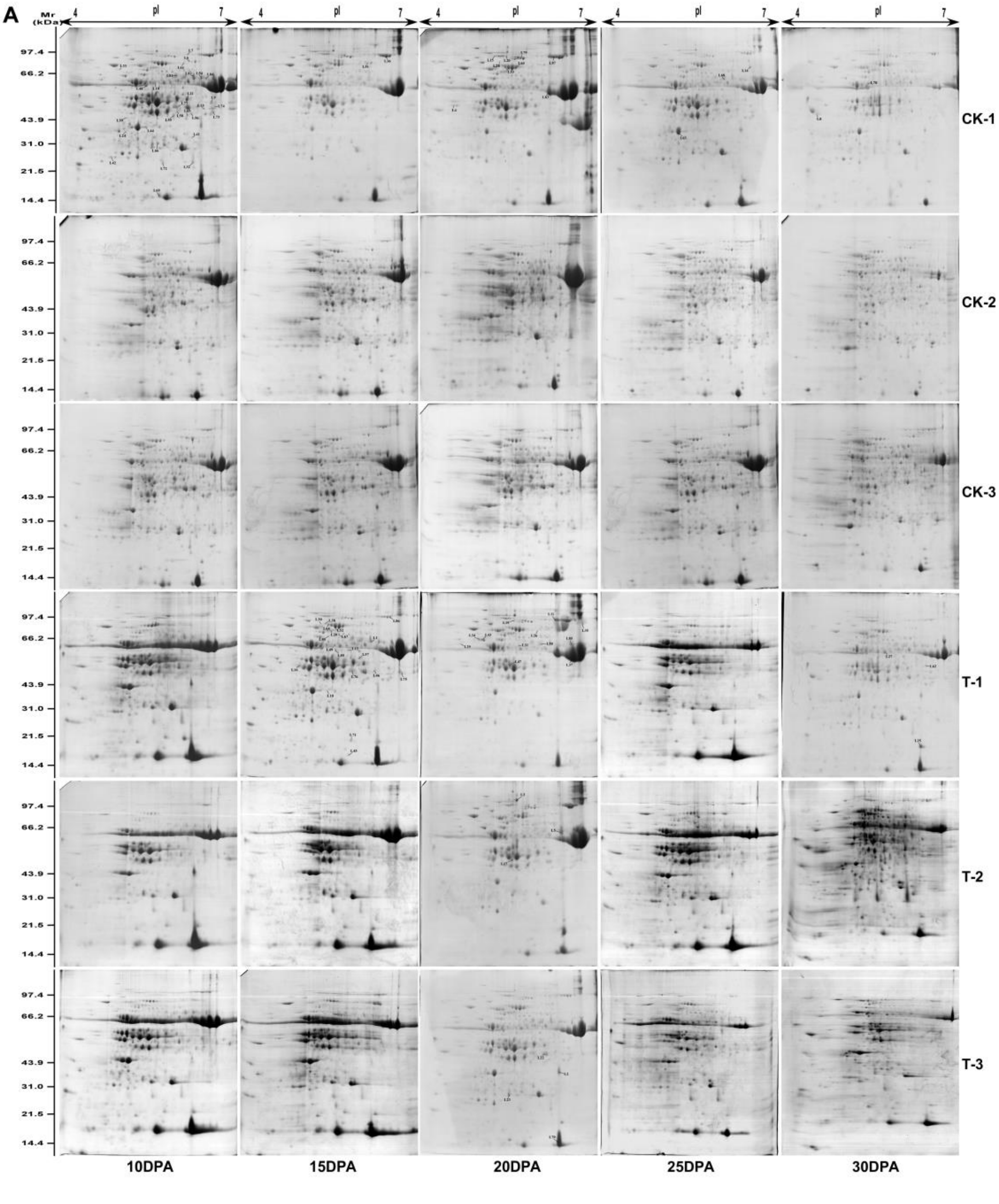
A
Mr (kDa)
4
pI
7
97.4
66.2
43.9
31.0
21.5
14.4
CK-1
CK-2
CK-3
T-1
T-2
T-3
10DPA
15DPA
20DPA
25DPA
30DPA

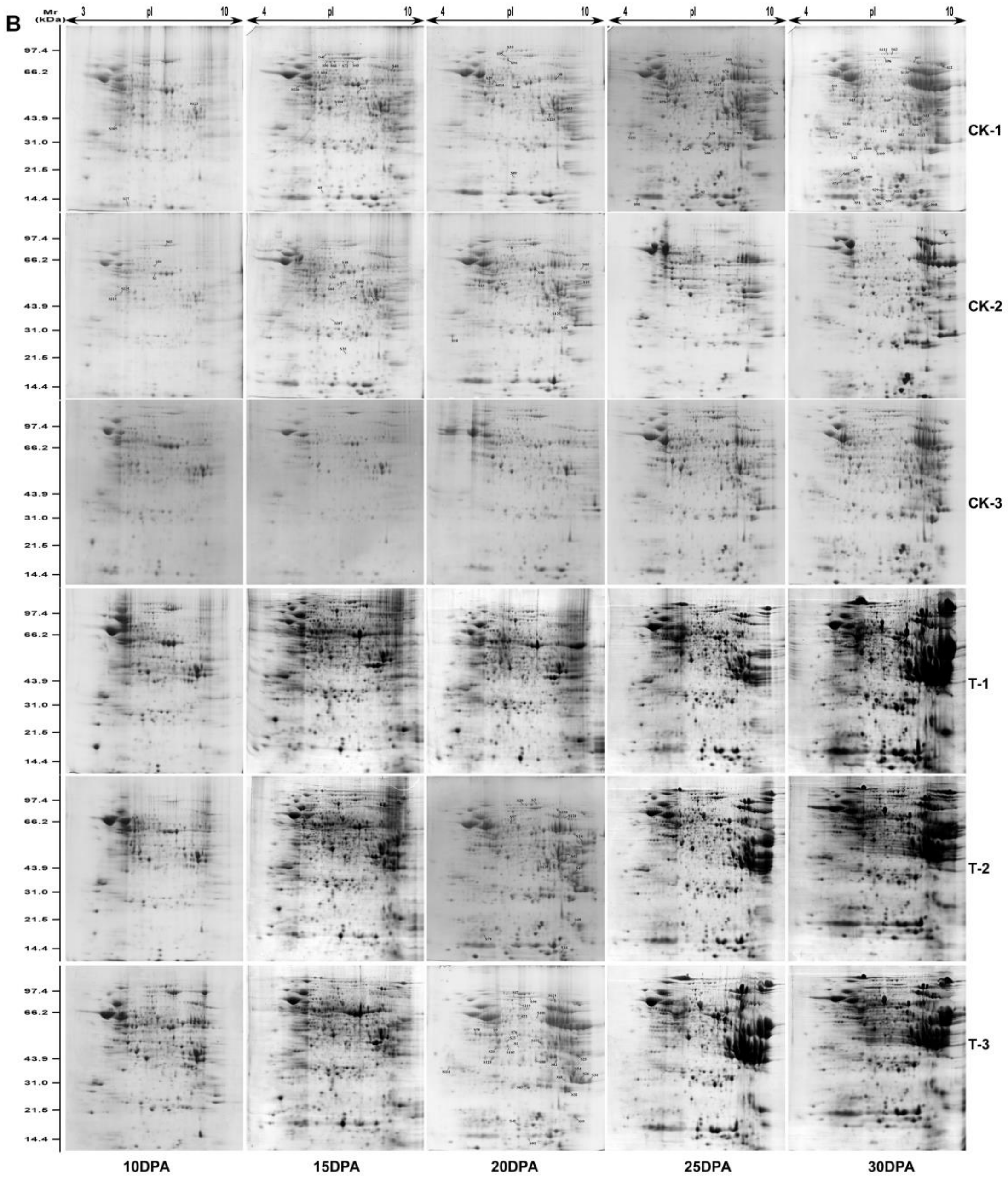

Figure S6. qRT-PCR analysis of representative DAPs in flag leaves and developing grains of Zhongmai 175. Statistically significant differences compared to the control were calculated based on an independent Student's t-tests: *$p < 0.05$; **$p < 0.01$.

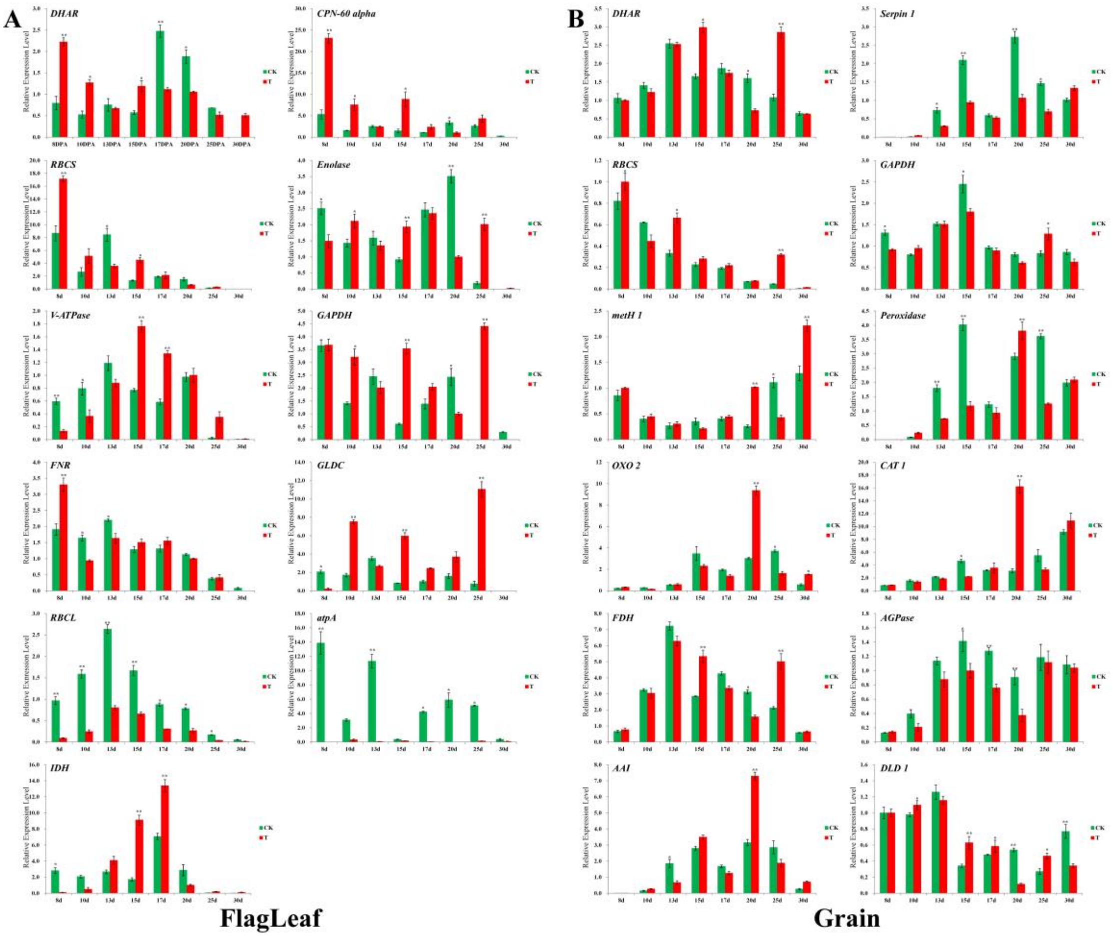

Figure S7. qRT-PCR optimal performance of standard curves and melting temperature curves. **(A)** Flag leaves. **(B)** Developing grains.

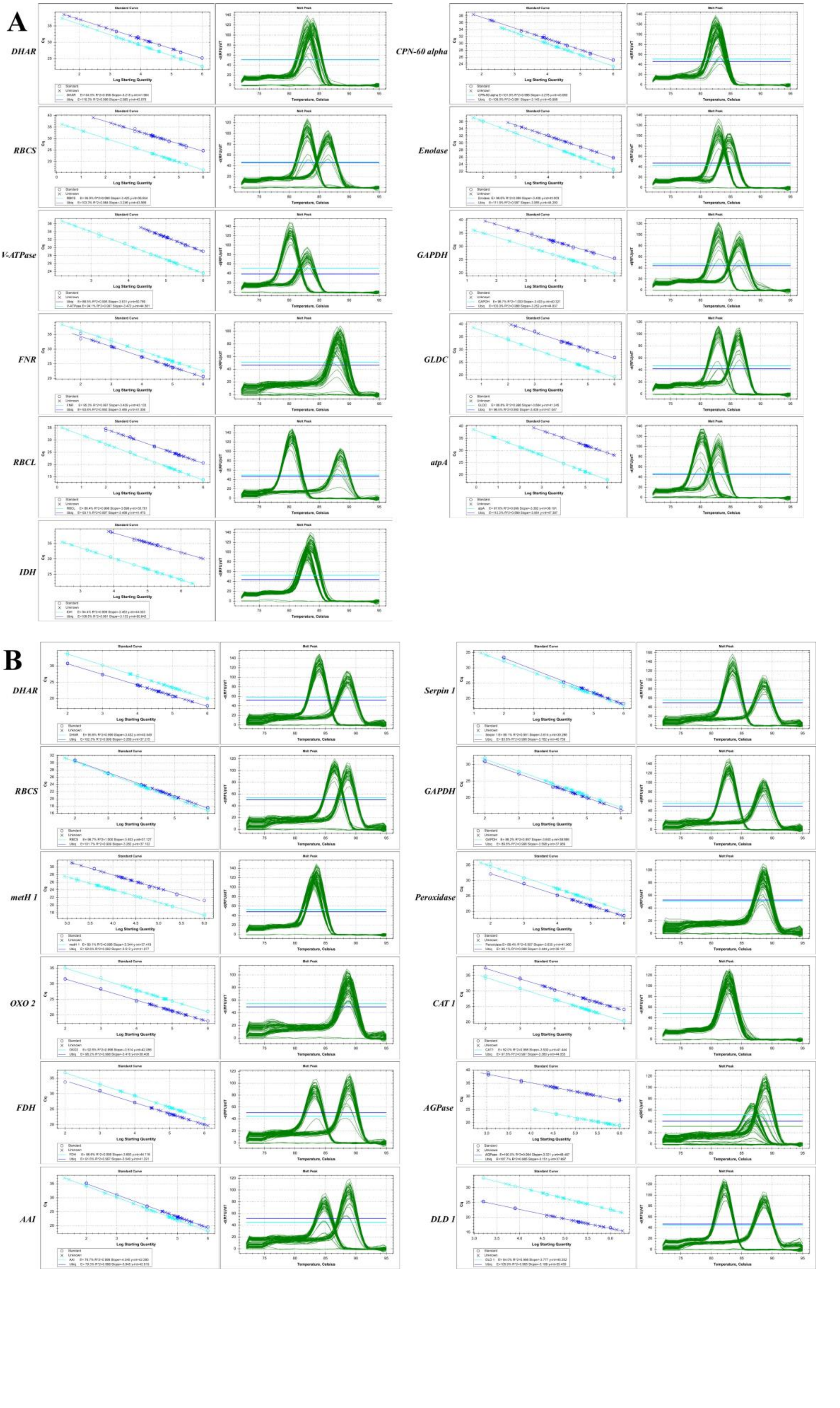
A
DHAR
CPN-60 alpha
RBCS
Enolase
V-ATPase
GAPDH
FNR
GLDC
RBCL
atpA
IDH
B
DHAR
Serpin 1
RBCS
GAPDH
metH 1
Peroxidase
OXO 2
CAT 1
FDH
AGPase
AAI
DLD 1
